# Severity of insomnia among counseling patients in psychiatry

**DOI:** 10.1192/j.eurpsy.2021.1481

**Published:** 2021-08-13

**Authors:** D. Falfel, W. Krir, H. Kefi, A. Omayya

**Affiliations:** Psychiatry, Military Hospital, Tunis, Tunisia

**Keywords:** prevalence, Insomnia, Insomnia Severity Index

## Abstract

**Introduction:**

Insomnia is a frequent reason of consultation in psychiatry. Always it is associates to other psychiatric pathologies.After stabilisation of the main disorder, it can become the only complaint.

**Objectives:**

This study aimed tob assess the prevalence of severe insomnia among patients suffering from different psychiatric disorder, and their sociodemographic profile.

**Methods:**

It is a cross sectional study conducted in February 2020 at the psychiatric ward of the military hospital of Tunis, including 80 patients who responded to the questionnaire of Insomnia Severity Index (ISI).

**Results:**

The study included 80 patients (18 to 66) years old with average age 38.78.The questionnaire showed that 26.92% didn’t have any sleep disorder, 25% had light insomnia, 42,30% had mild insomnia and only 5.76% suffered from severe insomnia.The patients counseling for anxiodepressive disorders were 48%, for PTSD were 17.46% and 17.3% for psychosis. Military population represented 80% of total patients interviewed and the average of years of service was17.7 years. The single patients were 46% th others were married. 70% of the patients were under hypnotic drugs besides the main treatment.

**Conclusions:**

Sleep disorders have a significant impact on cognitive functions and life quality which should be separately studied. Despite of well conducted pharmacotherapy, some patients still suffering from severe insomnia, it can be attributed to the main psychiatric disorder, relapse, treatment resistance, substance abuse…The importance of the psychiatrist involvement in screening and treatment can obviously enhance the prognosis. Other therapeutic alternatives which are non pharmacological such as phytotherapy ans CBT should be proposed to patients.

